# Sociodemographic factors influencing HIV transmission misconceptions among young Jordanian men: insights from the 2023 demographic and health survey

**DOI:** 10.1186/s12879-025-11756-y

**Published:** 2025-10-13

**Authors:** Ahmed Mohamed Shahin, Omar Abbas, Mahmoud Shaaban Abdelgalil

**Affiliations:** 1 Faculty of Medicine, Menoufia University, Menoufia, Egypt; 2 Faculty of Medicine, Ain-shams University, Cairo, Egypt; 3 Research Insights Arab Network, Cairo, Egypt

**Keywords:** HIV transmission misconceptions, HIV/AIDS stigma, Jordanian men, Sociodemographic determinants, Demographic and health surveys (DHS)

## Abstract

**Background:**

Misconceptions about HIV transmission, such as the belief that it spreads through sharing food, pose a challenge to public health education in Jordan. This study examines the demographic, socioeconomic, and media-related factors shaping these misconceptions among young Jordanian men.

**Methods:**

Using data from the 2023 Jordanian Demographic and Health Survey (JDHS), we conducted a univariate and multivariate logistic regression analysis to identify socioeconomic predictors of misconceptions about HIV transmission through food. The study utilized a nationally representative sample of 1,500 young Jordanian men aged 15–24 years. Participants were asked, “Can you get HIV by sharing food with a person who has AIDS?” Responses were categorized as “yes,” “no,” or “do not know.” Men with missing data or “do not know” responses were excluded. Data were weighted according to DHS guidelines to ensure national representativeness.

**Results:**

We included 1500 young Jordanian men, of which 445 (29.7%) thought that they could get HIV through sharing food with HIV patients. Our findings indicate that men with secondary education (AOR = 0.167, 95% CI: 0.061–0.457, *p* = 0.001) and higher education (AOR = 0.118, 95% CI: 0.039–0.355, *p* < 0.001) had significantly lower odds of holding misconceptions about HIV transmission. Media consumption patterns also influenced misconceptions. Frequent television viewing (AOR = 0.446, 95% CI: 0.276–0.720, *p* = 0.001) and internet usage (AOR = 0.493, 95% CI: 0.268–0.907, *p* = 0.023) were associated with reduced odds of misconceptions. In contrast, reading newspapers or magazines at least once a week (AOR = 2.820, 95% CI: 1.142–6.961, *p* = 0.025) and listening to the radio less than once a week (AOR = 1.770, 95% CI: 1.072–2.922, *p* = 0.026) were linked to higher odds of misconceptions. Additionally, men employed in household and domestic work exhibited a significantly increased likelihood of misconceptions (AOR = 57.975, 95% CI: 4.900–685.979, *p* = 0.001).

**Conclusion:**

This study underscores the need for targeted education and media strategies to address HIV misconceptions in Jordan, particularly among rural residents and those with lower education. Using accessible media like TV and improving health content on television can help dispel myths, reduce stigma, and foster a more informed society.

## Introduction

The human immunodeficiency virus (HIV) is a global health issue that primarily targets the human immune system, specifically white blood cells [1]. By the end of 2023, an estimated 39.9 million people were living with HIV worldwide, with HIV-related mortality reaching 630,000 [2].While HIV transmission rates vary across regions, countries such as Jordan face their own unique challenges. As of 2024, it is estimated that there are between 1,200 and 1,500 AIDS cases in Jordan, with approximately 55% of these cases attributed to unprotected, illegal sexual relationships [3, 4].

Despite significant advancements in public knowledge about HIV, misconceptions about its transmission continue to persist. Studies conducted in Kenya, Ghana, and Senegal have shown that some individuals mistakenly believe HIV can be transmitted through mosquito bites, witchcraft, or physical contact with an infected person [5, 6].These misconceptions contribute to stigma and discriminatory behaviors, including social avoidance, such as exclusion from sharing food, drinks, sitting nearby, or handling personal belongings. Such actions stem from widespread misunderstandings about HIV transmission [1].

In Jordan, although 97% of the population is aware of HIV and its modes of transmission, certain myths remain persistent and difficult to change [7]. One prevalent misconception is the belief that HIV can be transmitted through sharing food [8].The 2023 Jordanian Demographic and Health Survey (JDHS) found that 29.7% of young Jordanian men mistakenly believe that HIV can be transmitted through sharing food. The survey focused on young men, recognizing their increased risk due to shorter relationships, multiple partners, and other high-risk behaviors. Understanding the factors contributing to these misconceptions is essential for bridging knowledge gaps, guiding future research, and strengthening HIV prevention and control efforts [9–11].

This study aims to address these deeply ingrained misconceptions through a multivariate analysis, identifying the socioeconomic and cultural factors that drive such beliefs. By uncovering these determinants, the research will offer evidence-based insights that can guide culturally tailored public health interventions. These interventions are crucial in reducing misinformation and stigma surrounding HIV transmission, ultimately improving HIV prevention efforts in Jordan.

## Methods

### Data source

We extracted the necessary data from the 2023 JDHS. The survey included information on beliefs about HIV transmission. Our analysis focused on young men aged 15–24 years who responded to a specific question regarding the risk of contracting HIV through sharing food with individuals living with AIDS.

The question, as included in the database, was phrased as follows: “Can you get HIV by sharing food with a person who has AIDS?”. Exclusion criteria were men with missing data or those who responded “Do not know” for this question.

### Variables included

This study examined multiple variables, including age, wealth index, educational attainment, place of residence, occupation, and media consumption habits. Age was categorized into two groups: 15–19 years and 20–24 years. The wealth index was classified into five levels: poorest, poorer, middle, richer, and richest.

Educational attainment was categorized as no education, primary education, secondary education, or higher education. Place of residence was classified as either urban or rural. Occupation was assessed across multiple categories: not working, professional/technical/managerial, clerical, sales, self-employed in agriculture, household and domestic work, services, skilled manual labor, and unskilled manual labor.

Media consumption habits were assessed through four variables: frequency of using internet last month, frequency of listening to the radio, reading newspapers or magazines, and watching television. For each of these, responses were categorized as not at all, less than once a week, or at least once a week.

### Statistical analysis

The analysis was conducted using SPSS version 27. In line with the Demographic and Health Survey (DHS) guidelines [12], weighted counts were applied to ensure representativeness. The sample weight, an eight-digit number with six decimal places, was adjusted by dividing it by 1,000,000. Descriptive statistics were used to summarize the characteristics of the study population.

To identify predictors of the belief that HIV can be transmitted through sharing food, a logistic regression model was employed. Statistical analysis was conducted in two stages. First, univariate logistic regression was performed to identify potential predictors. Variables with a significance level of less than 0.2 in the univariate analysis were then included in the multivariate logistic regression model to determine independent associations [13]. Results from the logistic regression were reported using adjusted odds ratios (AORs) and their corresponding 95% confidence intervals (CIs). A p-value below 0.05 was deemed statistically significant.

Men who responded “yes” to the question were classified as the case group and coded as 1 in the DHS database, while those who responded “no” were classified as the control group and coded as 0. Men with missing data or those who responded “Do not know” were excluded from the analysis.

### Ethical consideration

Approval for data usage was granted by the DHS board on 28th October 2024. Before participating in the survey, informed consent was obtained from all participants. The consent process included a detailed explanation of the survey’s purpose, potential benefits, risks associated with participation, and the estimated duration of the survey. Participants were informed of their right to either agree or decline participation without any consequences.

## Results

### Characteristics of Jordanian men included in the analysis

For the analysis, a set of data was collected from 1,500 Jordanian men. Among them, 445 (29.7%) believed that they could get HIV by sharing food with someone who has AIDS **(**Table 1**)**. The results revealed that the highest proportion of men who believed in this misconception (case) were in the 15–19 years age group (52.4%), while the largest proportion of men who did not believe the misconception (control) were also in the same age group (50.4%).

**Table 1:** Sociodemographic characteristics of included Jordanian men in our analysis

		Can get HIV by sharing food with person who has AIDS			
		Control		Case	
		Count	Column *n* %	Count	Column *n* %
Age in 5-year groups	15–19	532	50.4%	233	52.4%
	20–24	523	49.6%	212	47.6%
Educational level	No education	6	0.6%	15	3.4%
	Primary	11	1.1%	13	2.9%
	Secondary	653	61.9%	297	66.7%
	Higher	385	36.5%	121	27.1%
Type of place of residence	Urban	987	93.6%	398	89.4%
	Rural	67	6.4%	47	10.6%
Wealth index combined	Poorest	113	10.7%	73	16.5%
	Poorer	136	12.9%	58	13.1%
	Middle	207	19.6%	91	20.5%
	Richer	267	25.3%	90	20.2%
	Richest	331	31.4%	133	29.8%
Frequency of reading newspaper or magazine	Not at all	1025	97.2%	411	92.2%
	Less than once a week	16	1.5%	12	2.8%
	At least once a week	14	1.3%	22	5.0%
	Almost every day	0	0.0%	0	0.0%
Frequency of listening to radio	Not at all	787	74.7%	274	61.5%
	Less than once a week	166	15.7%	103	23.1%
	At least once a week	101	9.6%	68	15.4%
	Almost every day	0	0.0%	0	0.0%
Frequency of watching television	Not at all	81	7.7%	56	12.5%
	Less than once a week	260	24.6%	131	29.3%
	At least once a week	713	67.6%	259	58.1%
	Almost every day	0	0.0%	0	0.0%
Frequency of using internet last month	Not at all	81	7.6%	74	16.5%
	Less than once a week	1	0.1%	7	1.6%
	At least once a week	21	2.0%	20	4.5%
	Almost every day	951	90.2%	345	77.3%
Occupation (grouped)	Not working	801	76.0%	351	78.7%
	Professional/technical/managerial	41	3.9%	15	3.3%
	Clerical	18	1.7%	2	0.5%
	Sales	32	3.0%	10	2.2%
	Agriculture - self employed	8	0.8%	1	0.2%
	Agriculture - employee	0	0.0%	0	0.0%
	Household and domestic	0	0.0%	3	0.7%
	Services	65	6.2%	25	5.6%
	Skilled manual	73	6.9%	26	5.8%
	Unskilled manual	17	1.6%	12	2.7%
	Don’t know	0	0.0%	2	0.4%

Regarding educational level, most case group had completed secondary education (66.7%), while 27.1% had higher education, and smaller proportions had primary (2.9%) or no education (3.4%). However, among control group 61.9% had completed secondary education, 36.5% had higher education, 1.1% had primary education, and 0.6% had no education. Approximately a quarter of the entire sample had no formal education. Regarding the wealth index combined, the highest proportion of the case and control group was from the richest wealth quintile (29.8%) and (31.4%) in order. More details about the baseline characteristics are reported in Table 1.

### Multivariable analysis

The multivariable analysis (Table 2) identified several significant predictors of the misconception that HIV can be transmitted by sharing food with someone who has AIDS. Men with secondary education (AOR = 0.167, 95% CI: 0.061–0.457, *p* = 0.001) and higher education (AOR = 0.118, 95% CI: 0.039–0.355, *p* < 0.001) were significantly less likely to believe in the misconception compared to those with no education. In contrast, men with primary education showed no significant association (AOR = 0.388, 95% CI: 0.064–2.346 *P* = 0.302).

**Table 2:** Predictors of beliefs about HIV transmission through food among Jordanian men

Parameter Estimates									
		Univariate analysis				Multivariate analysis			
		AOR	95% Confidence Interval for AOR			AOR	95% Confidence Interval for AOR		
			Lower	Upper	*P*- value		Lower	Upper	*P*- value
Age in 5-year groups	15-19	Reference				NA			
	20-24	0.924	0.632	1.351	0.684				
Educational level	No education	Reference				Reference			
	Primary	0.462	0.125	1.705	0.246	0.388	0.064	2.346	0.302
	Secondary	0.182	0.089	0.372	<.001	0.167	0.061	0.457	0.001
	Higher	0.126	0.059	0.269	<.001	0.118	0.039	0.355	<0.001
Type of place of residence	Urban	Reference				Reference			
	Rural	1.745	1.181	2.58	0.005	1.531	0.992	2.362	0.054
Wealth index combined	Poorest	Reference				Reference			
	Poorer	0.659	0.378	1.15	0.142	0.950	0.518	1.742	0.867
	Middle	0.680	0.371	1.247	0.212	1.171	0.596	2.299	0.646
	Richer	0.520	0.279	0.969	0.04	0.853	0.444	1.640	0.633
	Richest	0.619	0.349	1.095	0.099	1.247	0.669	2.326	0.487
Frequency of using internet last month	Not at all	Reference				Reference			
	Less than once a week	5.901	1.47	23.687	0.012	5.145	1.193	22.184	0.028
	At least once a week	1.046	0.465	2.356	0.913	1.008	0.416	2.443	0.985
	Almost every day	0.397	0.249	0.633	<.001	0.493	0.268	0.907	0.023
Frequency of reading newspaper or magazine	Not at all	Reference				Reference			
	Less than once a week	1.957	0.857	4.469	0.111	1.380	0.592	3.214	0.455
	At least once a week	4.105	2.159	7.807	<.001	2.820	1.142	6.961	0.025
Frequency of listening to radio	Not at all	Reference				Reference			
	Less than once a week	1.783	1.121	2.835	0.015	1.770	1.072	2.922	0.026
	At least once a week	1.942	1.031	3.658	0.04	2.007	0.962	4.190	0.063
Frequency of watching television	Not at all	Reference				Reference			
	Less than once a week	0.735	0.425	1.269	0.269	0.612	0.349	1.073	0.086
	At least once a week	0.530	0.341	0.825	0.005	0.446	0.276	0.720	0.001
Occupation (grouped)	Not working	Reference				Reference			
	Professional/technical/managerial	0.821	0.255	2.641	0.74	0.980	0.311	3.084	0.972
	Clerical	0.275	0.049	1.546	0.142	0.256	0.040	1.634	0.149
	Sales	0.702	0.295	1.671	0.424	0.699	0.293	1.669	0.420
	Agriculture - self employed	0.200	0.033	1.218	0.081	0.206	0.029	1.458	0.113
	Household and domestic	43.013	3.938	469.821	0.002	57.975	4.900	685.979	0.001
	Services	0.869	0.43	1.755	0.694	0.888	0.423	1.863	0.753
	Skilled manual	0.810	0.408	1.609	0.547	0.861	0.377	1.967	0.723
	Unskilled manual	1.659	0.503	5.466	0.405	1.448	0.471	4.452	0.517

Media consumption patterns were significant predictors of the misconception. Men who used the internet less than once a week had significantly higher odds of holding the misconception (AOR = 5.145, 95% CI: 1.193–22.184, *p* = 0.028). In contrast, those who used the internet daily had significantly lower odds (AOR = 0.493, 95% CI: 0.268–0.907, *p* = 0.023).

Similarly, men who read newspapers or magazines at least once a week were more likely to hold the misconception (AOR = 2.820, 95% CI: 1.142–6.961, *p* = 0.025), whereas reading less than once a week was not significantly associated (AOR = 1.380, 95% CI: 0.592–3.214, *p* = 0.455). Television viewing, on the other hand, showed a protective effect. Men who watched TV at least once a week were significantly less likely to hold the misconception (AOR = 0.446, 95% CI: 0.276–0.720, *p* = 0.001), while watching less than once a week had no significant impact (AOR = 0.612, 95% CI: 0.349–1.073, *p* = 0.086).

Regarding radio exposure, men who listened less than once a week had significantly higher odds of holding the misconception compared to those who never listened (AOR = 1.770, 95% CI: 1.072–2.922, *p* = 0.026). However, listening at least once a week was not significantly associated with the misconception (AOR = 2.007, 95% CI: 0.962–4.190, *p* = 0.063).

Regarding occupation, men working in household and domestic showed a significant increase in the odds of the misconceptions (AOR = 57.975, 95% CI: 4.900–685.979, *p* = 0.001). Other variables, such as age, wealth index, and type of place of residence did not show significant associations with the misconception.

## Discussion

This study focuses on the factors affecting misconceptions about HIV transmission in young Jordanian men, specifically the false belief that HIV can be transmitted by sharing food with someone who has AIDS. Our findings show that certain demographic factors and media habits are associated with this misconception, pointing to areas where targeted public health efforts might be effective in reducing HIV stigma and misinformation.

Our analysis found no significant association between age and misconceptions about HIV transmission through food, which aligns with findings from Seid et al. (2020) in Ethiopia [14] and Sano et al. (2016) in Malawi [15]. This contrasts with Mondal et al. (2015) in Bangladesh [16], which reported that older women were more likely to hold such misconceptions. The discrepancy between our study and Mondal et al. (2015) [16] could be attributed to differences in study populations and geographical contexts. Our study included men aged 15–24, and the lack of data for older age groups means we could not assess whether misconceptions vary in these populations. This limitation suggests that the relationship between age and HIV-related misconceptions may differ across age ranges and genders, warranting further investigation to better understand how misconceptions evolve across different demographic groups.

Education level has a strong impact. Individuals with secondary or higher education were less likely to believe in food-based HIV transmission. This pattern aligns with previous studies that show a higher level of education improves health knowledge and understanding of HIV transmission [17, 18]. This finding suggests that education provides people with the critical thinking skills and accurate information needed to eradicate HIV myths, underscoring the importance of integrating HIV education into schools [19, 20, 20–21].

Our analysis found no significant difference between living in rural versus urban areas regarding misconceptions about HIV transmission. This urban-rural divide contrasts with previous research indicating that rural populations typically have lower HIV awareness and higher levels of stigma compared to urban populations [21, 22]. The disparity is likely due to limited access to healthcare resources and educational programs in rural areas. Targeted public health initiatives in these regions could help address misconceptions and reduce stigma surrounding HIV.

Socioeconomic status, as measured by wealth, did not show a strong impact on HIV misconceptions. While those in the “richer” and “richest” categories showed a slightly higher impact on holding this belief, the differences were not significant. This finding is somewhat unexpected, as other studies have shown that wealthier individuals often have better health knowledge [15, 18]. It could be that wealth alone does not affect knowledge of HIV transmission, especially in areas where cultural beliefs and stigma may influence understanding more than economic factors.

The study’s findings on media consumption patterns provide valuable insights into the role of different media types in shaping HIV-related misconceptions. Surprisingly, men who read newspapers or magazines were more likely to hold the misconception. This contrasts with previous research suggesting that print media can effectively deliver health information and dispel myths [23]. One possible explanation is that the content of newspapers and magazines in Jordan may not consistently provide accurate health information, or that individuals who frequently read these materials may be exposed to sensationalized or misleading content.

Similarly, listening to the radio was associated with higher odds of holding the misconception. While radio is an effective tool for health education, particularly in rural areas [24], the study’s findings suggest that infrequent exposure to radio may not be sufficient to counteract misinformation. This highlights the need for more consistent and targeted radio programming that addresses HIV-related myths and provides accurate information.

In contrast, frequent television viewing was associated with lower odds of holding the misconception. This finding suggests that television may be a more effective medium for disseminating accurate health information, particularly when compared to print media and radio. However, it is important to note that not all television content is reliable, and some programs may inadvertently reinforce myths [25]. Public health campaigns should work with television networks to ensure that HIV-related content is scientifically accurate and culturally appropriate.

Moreover, the frequent use of internet was significantly associated with lower odds of holding the misconception. This may be attributed to the fact that the internet serves as a powerful tool for improving HIV knowledge and combating misconceptions [26–29]. It provides access to a vast array of health information, empowering individuals to better manage their condition and reduce vulnerability to misinformation [26–29]. Online platforms are effective in disseminating HIV-related information, which enhances knowledge and awareness, helping to correct existing myths. Additionally, online communities and social networking sites offer a space for users to ask questions and engage in informed discussions about HIV, which plays a key role in dispelling misconceptions [26–29].

Internet-based interventions, such as the Internet Popular Opinion Leaders (iPOL) program, have proven successful in increasing HIV/AIDS knowledge, boosting self-efficacy, and fostering positive behavior changes, including increased HIV testing and safer sexual practices. These initiatives contribute to reducing misconceptions by providing accurate, peer-supported information and promoting healthier attitudes and behaviors towards HIV [27].

Regarding occupation, our analysis found a significant increase in the odds of HIV misconception among men working in household and domestic. This may be attributed to limited access to formal education and health information. Individuals in these roles often have lower educational attainment, which has been linked to a higher likelihood of harboring misconceptions about HIV transmission.

### Strength and limitations

The key strength of our study lies in the use of nationally representative data from the 2023 Jordanian Demographic and Health Survey (JDHS), ensuring that our findings are grounded in the real-world experiences of 1,500 Jordanian men. The use of multivariate analysis allowed for the identification of key sociodemographic and media-related predictors of HIV misconceptions.

However, this study has several limitations that warrant further investigation in future research. First, its cross-sectional design limits causal inference, as it cannot establish temporal relationships between the misconception about HIV transmission through food and its associated risk factors. Second, self-reported media exposure may be subject to recall bias. Additionally, although we aimed to analyze all age groups, the dataset only included men aged 15–29, restricting our ability to assess misconceptions among older adults. The exclusion of women, older individuals, and respondents who answered “do not know” further limits the generalizability of our findings. Third, the study focused on a single misconception—HIV transmission through food-sharing—potentially overlooking broader HIV-related stigma dynamics. Lastly, the absence of qualitative data restricts the exploration of cultural and religious influences that may shape these misconceptions, which is particularly relevant in Jordan’s conservative social context.

### Recommendation

To combat the myth of HIV transmission through food, targeted public health interventions should focus on rural areas and individuals with lower education. Integrating HIV education into school curricula can enhance awareness among youth. Media campaigns should leverage radio’s reach in rural communities and ensure accurate televised health content. Community programs involving local leaders and culturally relevant materials can further dispel myths and reduce stigma. Future research should explore cultural influences on misconceptions and assess the effectiveness of different media formats in addressing them.

## Conclusion

This study highlights persistent misconceptions about HIV transmission among Jordanian young men, particularly the belief that HIV can spread through sharing food. Lower education levels, rural residence, and certain media consumption habits (e.g., reading newspapers/magazines and infrequent radio listening) were linked to higher odds of holding these misconceptions, while frequent TV viewing and internet usage was associated with lower odds. Targeted public health efforts should focus on improving education, especially in rural areas, and leveraging media to disseminate accurate HIV information. Addressing these factors can reduce stigma, correct misinformation, and enhance HIV prevention in Jordan. Future research should explore cultural influences and the effectiveness of media interventions in combating HIV myths.

## Data Availability

Data is available upon request from ICF International’s website (https://dhsprogram.com/data/available-datasets.cfm).
